# The influence of serum sterol sulfotransferase, ANGPTL8, and SDC1 on liver function in patients with intrahepatic cholestasis during pregnancy

**DOI:** 10.5937/jomb0-62650

**Published:** 2026-05-22

**Authors:** Qixin Wang, Ming Wang, Wenqi Wu

**Affiliations:** 1 The Affiliated People's Hospital of Ningbo University, Obstetrics and Gynaecology, Ningbo City, China; 2 The First Affiliated Hospital of Xuzhou Medical University, Department of Medical Laboratory, Xuzhou City, China

**Keywords:** intrahepatic cholestasis of pregnancy, Sterol Sulfotransferase, recombinant angiopoietin-like protein 8, proteoglycan-1, liver function, intrahepatična holestaza u trudnoći, sterol-sulfotransferaza, rekombinantni angiopoetinu sličan protein 8, proteoglikan-1, funkcija jetre

## Abstract

**Background:**

To explore the relationships between serum Sterol Sulfotransferase, Recombinant Angiopoietin Like Protein 8 (ANGPTL8), and Recombinant Syndecan (SDC1) and liver function in patients with intrahepatic cholestasis of pregnancy (ICP), as well as their influence on perinatal outcomes.

**Methods:**

The control group comprised 200 healthy pregnant women who underwent physical examinations during the same period, while the study group comprised 210 ICP patients admitted to our hospital between June 2023 and December 2024. The study group's and the control group's serum Sterol Sulfotransferase, ANGPTL8, SDC1, and liver function markers were compared. Pearson correlation analysis was used to assess the relationships between serum levels of Sterol Sulfotransferase, ANGPTL8, and SDC1 and various liver function indicators. The patients in the study group were divided into a poor-outcome group (86 patients) and a good-outcome group (124 patients) based on perinatal outcomes. Serum Sterol Sulfotransferase, ANGPTL8, and SDC1 levels were examined between the groups with negative and positive outcomes. Factors predicting unfavourable perinatal outcomes in patients with ICPs were examined using univariate and multivariate logistic regression analyses.

**Results:**

Despite lower serum Sterol Sulfotransferase levels, the study group had higher levels of ANGPTL8 and SDC1 than the control group. P &lt; 0 .0 5 indicated that the differences were statistically significant. The study group's levels of alkaline phosphatase (ALP), alanine aminotransferase (ALT), and aspartate aminotransferase (AST) were significantly higher than those of the control group (P&lt;0.05). The Pearson correlation analysis showed that while the levels of ANGPTL8 and SDC1 were positively associated with AST, ALT, and ALP (P&lt; 0.05), the level of serum Sterol Sulfotransferase was negatively associated with these levels. Compared to the group that experienced a positive outcome, the unfavourable outcome group's serum Sterol Sulfotransferase level was lower, whereas SDC1 and ANGPTL8 levels were greater than those in the group with favourable results. Decreased serum Sterol Sulfotransferase levels (S23 mmol/L), increased ANGPTL8 levels (650 pg/mL), and poor perinatal outcomes were associated with elevated SDC1 levels (53 ng/mL) in patients with ICP (P&lt;0.05).

**Conclusions:**

Serum Sterol Sulfotransferase, ANGPTL8 and SDC1 are closely related to liver function and perinatal outcomes in patients with ICP. As the level of serum Sterol Sulfotransferase decreases and the levels of ANGPTL8 and SDC1 increase, it can lead to aggravated liver function impairment in patients with ICP, and it leads to adverse perinatal outcomes.

## Abstract (sr)

**Uvod:**

Cilj e ispita povezanost serumske sterol-sulfotransferaze, rekombinantnog angiopoetinu sličnog proteina 8 (ANGPTL8) i rekombinantnog sindekana-1 (SDC1) sa funkcijom jetre kod pacijentkinja sa intrahepatičnom holestazom u trudnoći (ICP), kao i njihov uticaj na perinatalne ishode.

**Metode:**

Kontrolnu grupu je činilo 200 zdravih trudnica koje su u istom periodu imale rutinske preglede, dok je ispitivanu grupu činilo 210 pacijentkinja sa ICP-om primljenih u našu ustanovu između juna 2023. i decembra 2024. Upoređeni su serumski nivoi sterol-sulfotransferaze, ANGPTL8, SDC1 i parametri funkcije jetre između grupa. Pearsonova korelaciona analiza korišćena je za procenu povezanosti nivoa serumske sterol-sulfotransferaze, ANGPTL8 i SDC1 sa indikatorima funkcije jetre. Pacijentkinje su podeljene na grupu sa lošim ishodom (86 pacijentkinja) i grupu sa dobrim ishodom (124 pacijentkinja) prema perinatalnim ishodima. Upoređeni su nivoi sterol-sulfotransferaze, ANGPTL8 i SDC1 između grupa sa povoljnim i nepovoljnim ishodima. Prediktori nepovoljnih perinatalnih ishoda kod ICP su analizirani univarijantnom i multivarijantnom logističkom regresijom.

**Rezultati:**

Ispitivana grupa je imala niži nivo serumske sterol-sulfotransferaze, a više nivoe ANGPTL8 i SDC1 u poređenju sa kontrolnom grupom (P &lt; 0 ,0 5 ). Nivoi alkalne fosfataze (ALP), alanin-aminotransferaze (ALT) i aspartat-aminotransferaze (AST) bili su značajno viši u ispitivanoj grupi (P&lt;0.05). Pirsonova analiza je pokazala da su nivoi ANGPTL8 i SDC1 pozitivno korelisani sa nivoima AST, ALT i ALP (P &lt; 0 ,0 5 ), dok je nivo serumske sterol-sulfotransferaze negativno korelisan sa ovim parametrima. Kod pacijentkinja sa nepovoljnim ishodom zabeležen je niži nivo sterol-sulfotransferaze, a viši nivoi SDC1 i ANGPTL8 u poređenju sa pacijentkinjama sa povoljnim ishodom. Smanjeni nivoi sterol-sulfotransferaze (S23 mmol/L), povišeni nivoi ANGPTL8 (S650 pg/mL) i povišeni nivoi SDC1 (S53 ng/mL) bili su povezani sa nepovoljnim perinatalnim ishodima kod ICP (P&lt;0 ,0 5 ).

**Zaključak:**

Nivoi serumske sterol-sulfotransferaze, ANGPTL8 i SDC1 blisko su povezani sa funkcijom jetre i perinatalnim ishodima kod pacijentkinja sa ICP. Smanjeni nivo serumske sterol-sulfotransferaze, uz istovremeno povećane nivoe ANGPTL8 i SDC1, može dovesti do pogoršanja oštećenja funkcije jetre i do nepovoljnih perinatalnih ishoda kod pacijentkinja sa ICP.

## Introduction

One of the most frequent side effects of idiopathic pregnancy is intrahepatic cholestasis of pregnancy (ICP) [1]. Its clinical signs include skin itching, and higher levels of serum alanine aminotransferase (ALT) and total bile acids are the leading indicators of liver function impairment. Premature birth, fetal discomfort in utero, neonatal death, etc., are all extremely potential outcomes if no proactive and successful intervention is offered [2]. At present, controversy persists regarding the pathogenesis of ICP, which may be related to genetics, endocrine disorders, abnormal immune function, and other factors. Sterol Sulfotransferase is an indispensable "catalyst" in the normal operation of the liver, plays a key role in the sulfation of bile acids, promotes the excretion of bile acids, and is related to cholestasis [3][4][5]. ANGPTL8 is a secretory protein that can mediate lipid metabolism processes by influencing pancreatic [3-cell function and lipoprotein enzyme levels, and further affects ICR Recombinant Syndecan (SDC1) can regulate processes such as cell growth, differentiation and adhesion by connecting the cellular microenvironment and cytoskeleton, thereby affecting liver function and possibly mediating the occurrence and development of ICP [6].

Pregnant women who have intrahepatic cholestasis of pregnancy (ICP), a common liver illness, may experience jaundice, cholestasis, and abnormal liver function [7]. The occurrence of ICP is related to multiple factors, including changes in hormone levels during pregnancy, genetic susceptibility, and environmental factors [8]. Studies [9][10][11] have shown that ICP may lead to fatal distress, premature birth and even stillbirth. Thus, early diagnosis and efficient treatment are essential for protecting mothers' and babies' health. In recent years, biomarkers such as Sterol Sulfotransferase, ANGPTL8 and SDC1 have gradually attracted increasing attention in ICP research. Sterol Sulfotransferase, a liver metabolic enzyme, is closely related to bile acid metabolism. ANGPTL8 is related to the regulation of fat metabolism and insulin secretion [12]. SDC1 plays an important role in cellular signal transduction and the formation of the extracellular matrix. These factors may play important roles in patients with ICP, influencing liver function and its metabolic regulatory mechanism [13][14][15].

By detecting serum levels of these biomarkers, their potential roles in ICP can be explored and their relationship with liver function impairment evaluated, thereby providing a new theoretical basis and clinical guidance for the early diagnosis and treatment of ICP.

## Materials and methods

### General information

The study group comprised 210 ICP patients hospitalised at our hospital between June 2023 and December 2024. An additional 200 healthy pregnant mothers who underwent physical examinations during the same time period comprised the control group. Participants in the research group were between 20 and 39 years old, with an average age of 27.41±2.34 years. There were 152 primiparas and 58 multiparas. The body mass index ranged from 18 to 32 kg/m^2^, with an average of 22.62±2.07 kg/m^2^. The gestational age ranged from 13 to 38 weeks, with an average of 29.88±0.65 weeks. The age of the individuals in the control group ranged from 20 to 40 years, with an average age of 27.58±2.39 years. The body mass index ranged from 18 to 32 kg/m^2^, with an average of 22.79±2.02 kg/m^2^. With an average of 29.76±0.73 weeks, the gestational age varied from 14 to 37 weeks. The general data did not show any statistically significant changes in age, body mass index, or gestational weeks.

### Inclusion criteria and exclusion criteria

Inclusion criteria: (1) All patients with ICP met the ICP-related diagnostic criteria formulated by the Obstetrics Group of the International Branch of Obstetrics and Gynaecology. (2) Age 18 years old; (3) No relevant treatment had been received before enrolment.

Exclusion criteria: (1) Combined with haematological diseases or/and other metabolic diseases; (2) Accompanied by malignant tumours; (3) Previous history of hepatitis or liver cirrhosis; (4) Withdrawal due to certain reasons during the research period.

### Research methods

(1) Sample collection: 4 millilitres of fasting venous blood were drawn on the day of the physical examination for healthy pregnant women, and after all patients were admitted. The blood was centrifuged for ten minutes at 3,000 rpm. The serum was centrifuged, then placed in an EP test tube and stored at -80°C for analysis.

(2) Serum index detection: Using an AU5800 completely automatic biochemical analyser, the levels of serum Sterol Sulfotransferase, ANGPTL8, and SDC1 were measured using an enzyme-linked immunosorbent assay (Beckman Coulter, USA). The specific operation was completed according to the instructions provided with the kit (Shanghai Lanji Biotechnology Co., Ltd.).

(3) Liver function index detection: A Hitachi 7060 completely automatic biochemical analyser detects the primary markers, which are alkaline phosphatase (ALP), alanine aminotransferase (ALT), and aspartate aminotransferase (AST).

(4) All research subjects were instructed to receive regular foetal heart rate monitoring at the hospital. Maturation promotion treatment was considered based on the disease progression and gestational weeks of the research subjects, and caesarean section or vaginal delivery was considered according to their specific conditions. Preterm birth, stillbirth, foetal distress in utero, foetal growth retardation in utero and neonatal asphyxia were regarded as adverse perinatal outcomes.

### Laboratory testing methods

All serological indicators in this study were detected in accordance with strict standard operating procedures. Serum samples were collected at enrolment, centrifuged, and frozen at -80°C until batch testing. The serum concentrations of Sterol Sulfotransferase, ANGPTL8, and SDC1 were measured by a double-antibody sandwich enzyme-linked immunosorbent assay (ELISA). Serum Sterol Sulfotransferase levels were measured using Cloud-Clone Corp from the United States. The human sulfotransferase Family 2A member 1 (Sterol Sulfotransferase) ELISA kit produced was used for quantitative detection, with the product number SEB674Hu. Serum ANGPTL8 (also known as ANGPTL8 or TD26) was determined using the Human angiopoietin-like protein 8 (ANGPTL8) ELISA kit from Wuhan Huamei Bioengineering Co., LTD. (Cusabio), with the product number CSB-EL009310HU. The concentration of serum SDC1 (CD138) was measured using the human SDC1 ELISA kit from Diaclone SAS (France; item number 850.630.096). All ELISA experiments were carried out strictly according to the instructions for each kit. Finally, the absorbance (OD) at 450 nm was measured on a microplate reader (e.g., SpectraMax M3, Molecular Devices), and the concentration of the corresponding marker in the sample was calculated from the standard curve. Detection of liver function indicators was performed on the routine biochemical analysis platform of our hospital's laboratory, using standard reagents and methods compatible with this platform. The specific test items include alanine aminotransferase (ALT), aspartate aminotransferase (AST), total bilirubin (TBil), direct bilirubin (DBil), total bile acids (TBA), alkaline phosphatase (ALP), γ-glutamyltransferase (GGT), and albumin (Alb). All testing processes are equipped with quality control samples to ensure the accuracy and reliability of the results, and are carried out by experienced inspection technicians. All samples undergo re-well testing.

### Statistical analysis

Data analysis and processing were conducted using SPSS 22.0. The independent sample t-test, sometimes called the corrected t-test, was used to compare the two groups. The measurement data, given as x̄±s, follow a normal distribution. Groups were compared using the χ^2^ test, and counts are reported as percentages or as the total number of cases. Pearson correlation analysis was used to examine the relationships between the levels of serum Sterol Sulfotransferase, ANGPTL8, and SDC1 and various liver function indicators. The factors predicting unfavourable perinatal outcomes in ICP patients were examined using univariate and multivariate logistic regression analyses, with a significance level of α = 0.05.

## Results

### Comparison of the serum Sterol Sulfotransferase, ANGPTL8 and SDC1 levels between the study group and the control group

The study group had a lower serum Sterol Sulfotransferase level than the control group. However, compared to the control group, the levels of SDC1 and ANGPTL8 were higher. The differences were statistically significant when P<0.05; see Table 1.

**Table 1:** Comparison of serum levels of Sterol Sulfotransferase, ANGPTL8, and SDC1 between the study group and the control group.

Group	n	Sterol Sulfotransferase (mmol/L)	ANGPTL8 (pg/mL)	SDC1 (ng/mL)
Research group	210	23.41±3.75	650.40±72.33	53.03±6.21
Control group	200	45.69±6.81	551.96±56.22	22.66±4.19
t		28.515	10.910	41.004
P		<0.001	<0.001	<0.001

There were significant differences in the levels of three markers in the serum of patients with intrahepatic cholestasis of pregnancy (ICP) compared with healthy pregnant women. Specifically, the serum Sterol Sulfotransferase levels in the study group were significantly lower than those in the healthy control group. On the contrary, the levels of serum ANGPTL8 (i.e. ANGPTL8) and SDC1 in the study group were significantly higher than those in the healthy control group. The differences between the above groups were all statistically significant (P<0.05). These results clearly indicate that in ICP patients, the expression of serum Sterol Sulfotransferase shows a down-regulated trend, while the expressions of ANGPTL8 and SDC1 show a significant up-regulated trend.

### Comparison of liver function index levels between the study group and the control group

The study group's AST, ALT, and ALP levels were all considerably higher than the control group's (P<0.05), as indicated in Table 2.

**Table 2:** Comparison of liver function indicators between the study group and the control group.

Group	n	AST (U/L)	ALT (U/L)	ALP (U/L)
Research group	210	84.64±12.33	78.28±18.46	136.85±22.37
Control group	200	16.23±3.14	10.33±2.14	49.08±10.10
t		55.173	37.525	36.487
P		<0.001	<0.001	<0.001

The liver function indicators of patients with intrahepatic cholestasis of pregnancy (ICP) were significantly abnormal compared with those of healthy pregnant women. The levels of serum alanine aminotransferase (ALT), aspartate aminotransferase (AST), and alkaline phosphatase (ALP) in the study group were significantly higher than those in the healthy control group, and these differences were statistically significant (P<0.05).

In addition, key indicators reflecting cholestasis status, such as serum total bilirubin (TBil), direct bilirubin (DBil), and total bile acid (TBA) levels, also showed a significant upward trend among patients in the study group. There was no significant statistical difference in serum albumin (Alb) levels between the two groups. This series of observations clearly indicates that patients with ICP generally have obvious hepatocyte damage and bile excretion dysfunction. Their liver biochemical indicators show characteristic changes, highly consistent with the disease essence of ICP (intrahepatic cholestasis) and the hepatocyte damage it causes.

### Correlation analysis of the serum Sterol Sulfotransferase, ANGPTL8, and SDC1 levels and the levels of various liver function indicators

According to the Pearson correlation analysis, there was a negative association (P<0.05) between serum Sterol Sulfotransferase levels and AST, ALT, and ALP The levels of ANGPTL8 and SDC1 were positively correlated with those of AST, ALT, and ALP (P<0.05; see Table 3).

**Table 3:** Correlation analysis between serum levels of Sterol Sulfotransferase, ANGPTL8, SDC1 and various liver function indicators.

Liver function<br>indicators	Sterol Sulfotransferase		ANGPTL8		Syndecar-1	
	r	P	r	P	r	P
AST	-0.527	<0.001	0.474	0.005	0.564	<0.001
ALT	-0.534	<0.001	0.535	<0.001	0.434	0.007
AIP	-0.502	<0.001	0.519	<0.001	0.405	0.016

The level of serum Sterol Sulfotransferase was significantly negatively correlated with the indicators reflecting liver cell damage - aspartate aminotransferase (AST), alanine aminotransferase (ALT), and the indicator reflecting cholestasis-alkaline phosphatase (ALP). Conversely, both serum ANGPTL8 and SDC1 levels showed significant positive correlations with AST, ALT, and ALP In addition, the increase in these two markers is significantly positively associated with the rise in total bile acid (TBA) levels, an important indicator of cholestasis severity.

### Univariate analysis of adverse perinatal outcomes

The patients in the study group were divided into a poor-outcome group (86 patients) and a good-outcome group (124 patients) based on perinatal outcomes. According to univariate analysis, while the ANGPTL8 and SDC1 levels were significantly higher (P<0.05) than those of the excellent outcome group, the blood Sterol Sulfotransferase level was lower in the bad outcome group. However, age, body mass index, gestational age, and number of deliveries were not associated with adverse perinatal outcomes in ICP patients (P>0.05; see Table 4).

**Table 4:** Univariate analysis of adverse perinatal outcomes.

Group	n	Age<br>(years)	Body Mass<br>Index<br>(kg/m^2^)	Gestational<br>age (week)	Production frequency		Sterol<br>Sulfotransferase<br>(mmol/L)	ANGPTL8<br>(pg/mL)	SDC1<br>(ng/mL)
					0 times	1 time			
Adverse<br>outcome<br>group	86	27.90±2.54	23.04±2.02	29.72±0.50	56<br>(65.12)	30<br>(34.88)	17.41±3.13	705.21±80.48	60.37±7.25
Good<br>outcome<br>group	124	27.17±2.18	22.40±2.03	29.93±0.67	96<br>(77.42)	28<br>(22.58)	27.68±4.58	612.48±66.74	47.94±5.74
t/χ^2^		1.812	1.332	0.908	1.925	12.743	6.444	9.837	
P		0.078	0.188	0.361	0.169	<0.001	<0.001	<0.001	

### The variables impacting unfavourable perinatal outcomes in patients with ICP by multivariate logistic regression

An unconditional multivariate logistic regression model was established: the levels of blood Sterol Sulfotransferase, ANGPTL8, and SDC1 were utilised as independent variables, while the perinatal outcome of ICP patients was used as the dependent variable (adverse outcome = 1, good result = 0). Given that the sample size is not too large, to achieve robust regression, the continuous numerical indicators among the independent variables to be included are transformed into binary classifications. According to clinical practice or custom, the cut-off value for transformation is the mean or median of all samples in the unfavourable and good outcome groups, and it is suitably rounded. Regression was performed using the stepwise method (α in = 0.05, α out = 0.10). The results of regression analysis revealed that decreased serum Sterol Sulfotransferase levels (≤23 μmol/L), increased ANGPTL8 levels (650 pg/mL), and elevated levels of SDC1 (53 ng/mL) were risk factors for unfavourable perinatal outcomes in ICP patients (P<0.05) (Table 5).

**Table 5:** Analysis of the determining factors for unfavourable perinatal outcomes in ICP patients by multivariate logistic regression.

Indicator	Assignment Design	β	SE	Wald X2	P	OR	95% CI
Constant	-	-0.174	0.085	4.346	0.030	-	-
Sterol Sulfotransferase	≤23 μmol/L=1, >23 mmol/L=0	0.605	0.181	10.261	0.001	1.828	1.266~2.632
ANGPTL8	650 pg/mL=1, <650 pg/mL=0	0.362	0.149	6.413	0.014	1.440	1.080~1.927
SDC1	53 ng/mL=1, <53 ng/mL=0	0.452	0.120	13.095	<0.001	1.585	1.237~2.022

## Discussion

ICP is more common in pregnant women in the middle and late stages [16]. Currently, it is generally believed to be related to disorders of glucose and lipid metabolism in patients [17]. Patients with ICP often have varying degrees of cholestasis, which makes it impossible for them to complete the hepatointestinal circulation smoothly, resulting in abnormal secretion of insulin growth factor-1, further causing disorders in the normal function of β cells and ultimately leading to disorders in glucose metabolism. In addition, after ICP occurs, the high accumulation of bile in the liver causes direct damage to liver cells and tissues, thereby affecting the quality of life and health of mothers and infants [18]. Relevant studies [19][20][21] have shown that abnormally elevated total bile acid levels are reliable indicators for effectively evaluating ICP. Moreover, specifically elevated total bile acid levels in ICP patients can lead to intrauterine hypoxia in newborns, which in turn can lead to a series of adverse neonatal outcomes. However, patients with other hepatobiliary diseases also have abnormal changes in total bile acid levels, which limits the specificity of this indicator in determining the neonatal outcome of ICP Serological index detection is a detection technology that has attracted much attention in recent years [22]. It can assist in diagnosing various diseases and offers the advantages of simple operation, low invasiveness, and high repeatability. Therefore, it is imperative to identify more valuable ICP evaluation indicators.

While SDC1 and ANGPTL8 levels were higher in the study group than in the control group, the study group's serum Sterol Sulfotransferase level was lower. This finding reflects the abnormally low expression of serum Sterol Sulfotransferase in ICP patients. However, the levels of serum ANGPTL8 and SDC1 were significantly high. Analysis of the cause revealed that Sterol Sulfotransferase is an important member of the sulfotransferase family that mediates the sulfation process of steroid hormones in the body. When its expression level decreases, it may be unable to increase the water solubility of bile acids, thereby blocking their excretion, leading to bile acid accumulation in the body and liver cell damage, ultimately causing ICP ANGPTL8 is an angiogenic-like protein secreted by the liver and adipose tissue. Its main functions are to regulate lipid metabolism and improve insulin resistance [23]. After ICP occurs, the body initiates a compensatory mechanism that increases ANGPTL8 levels. SDC1 is a transmembrane cell-surface molecule that can initiate coupling between the extracellular and intracellular environments and plays a crucial role in intertissue signal transduction [24]. It indirectly mediates liver injury by regulating pathways involved in hepatocyte proliferation and apoptosis [25]. Compared with total bile acid levels, the levels of the aforementioned serological indicators are less affected by other liver diseases, thereby potentially achieving greater diagnostic specificity. AST, ALT, and ALP values in the study group were all higher than those in the control group, indicating that patients with ICP clearly had impaired liver function [26].

After ICP occurs, it leads to significant bile stasis in the liver, which further causes direct liver damage. According to Pearson correlation analysis, there was a negative association between the levels of AST, ALT, and ALP and the serum Sterol Sulfotransferase level, whereas the levels of ANGPTL8 and SDC1 were positively correlated with those of AST, ALT, and ALP These findings suggest that serum levels of Sterol Sulfotransferase, ANGPTL8, and SDC1 are associated with liver function in ICP patients [27][28][29][30]. That is, as the level of serum Sterol Sulfotransferase decreases and the levels of ANGPTL8 and SDC1 increase, the liver function of ICP patients worsens. The analysis of the cause revealed that Sterol Sulfotransferase is one of the key enzymes in bile acid synthesis in the liver. When the level of Sterol Sulfotransferase is too low, it is difficult for it to sulfate bile acids, thus failing to promote their formation as primary binding bile acids in the body [31][32][33]. This leads to increased bile acid levels and intensifies liver cell toxicity. ANGPTL8 can inhibit lipoprotein lipase activity, thereby increasing triglyceride levels, promoting the development of metabolic disorders, and further aggravating liver damage in patients [34].

ANGPTL8 and SDC1 levels were higher in the adverse end group than in the good outcome group; however, the serum Sterol Sulfotransferase level was lower in the adverse end group than in the good outcome group, according to the study's findings [35]. The possible cause is abnormal expression of the above indicators, which can damage the patient's liver tissue by regulating bile acid levels, inhibiting lipoprotein lipase activity, and suppressing transforming growth factor-β1, and can also have an indirect impact on the foetus [36]. Decreased serum Sterol Sulfotransferase levels (≤23 μmol/L), increased ANGPTL8 levels (650 pg/mL), and elevated SDC1 levels ( 53 ng/mL) were risk factors for unfavourable perinatal outcomes in ICP patients.

## Conclusion

The expression of serum Sterol Sulfotransferase, ANGPTL8, and SDC1 is aberrant in ICP patients, is associated with liver function and perinatal outcomes, and can serve as a reliable indicator for the clinical auxiliary diagnosis of ICP.

## Dodatak

### Funding

Ningbo Health Science and Technology Program (No. 2022y39).

### Ethical approval

2023-(Scientific research)-19.

### Conflict of interest statement

All the authors declare that they have no conflict of interest in this work.

## References

[1] Tang M, Xiong L, Cai J, Fu J, Liu H, Ye Y, Yang L, Xing S, Yang X (2024;Feb). Intrahepatic cholestasis of pregnancy: Insights into pathogenesis and advances in omics studies. Hepatol Int.

[2] Liu Y, Wei Y, Chen X, Huang S, Gu Y, Yang Z, Guo X, Zheng H, Feng H, Huang M, Chen S, Xiao T, Hu L, Zhang Q, Zhang Y, Chen G, Qiu X, Wei F, Zhen J, Liu S (2025;May). Genetic study of intrahepatic cholestasis of pregnancy in Chinese women unveils East Asian etiology linked to historic HBV epidemic. J Hepatol.

[3] Tai M, Chen L, He Y, Wang F, Tian Z (2024;Jan 2). Ultrasonographic evaluation of the gallbladder motor function in the diagnosis and prognosis of intrahepatic cholestasis of pregnancy. BMC Pregnancy Childbirth.

[4] Huang H, Gao J, Dong R, Wang R, Li L, Wang G, Shi Y, Luo K, Chen J, Yuan W, Tian X, Zhao H, Zhang T (2025;Jan 3). Detection of serum lactate dehydrogenase A and its metabolites on placental function in patients with intrahepatic cholestasis of pregnancy. Int Immunopharmacol.

[5] Zeng W, Hou Y, Gu W, Chen Z (2024;Jun). Proteomic Biomarkers of Intrahepatic Cholestasis of Pregnancy. Reprod Sci.

[6] Iqbal M, Muhammad Z, Akhter N, Shams A S (2024;Oct 3). Effects of Ursodeoxycholic Acid Treatment for Intrahepatic Cholestasis of Pregnancy on Maternal and Fetal Outcomes. Cureus.

[7] Xiong L, Tang M, Xing S, Yang X (2023;Oct 13). The role of noncoding RNA and its diagnostic potential in intrahepatic cholestasis of pregnancy: A research update. Front Genet.

[8] Valdovinos-Bello V, García-Romero C S, Cervantes-Peredo A, García-Gómez E, Martínez-Ibarra A, Vázquez-Martínez E R, Valdespino Y, Cerbón M (2023;Jan-Feb). Body mass index implications in intrahepatic cholestasis of pregnancy and placental histopathological alterations. Ann Hepatol.

[9] Jasak K, Gajzlerska-Majewska W, Jabiry-Zieniewicz Z, Litwińska-Korcz E, Litwińska M, Ludwin A, Szpotańska-Sikorska M (2025;Aug 10). Intrahepatic Cholestasis of Pregnancy: Diagnosis, Management, and Future Directions: A Review of the Literature. Diagnostics (Basel).

[10] Arslanoğlu T, Bilirer K K, Demirkıran C İ, Ceylan Y, Veliyeva S, Koç İ N, Polat İ (2025;Apr 25). Intrahepatic cholestasis of pregnancy and coagulation: A dual risk of hypercoagulability and bleeding. BMC Pregnancy Childbirth.

[11] Huang X, Gu H, Shen P, Zhang X, Fei A (2024;Jun 4). Systematic review and meta-analysis: Evaluating the influence of intrahepatic cholestasis of pregnancy on obstetric and neonatal outcomes. PLoS One.

[12] Wu L, Zheng Y, Liu J, Luo R, Wu D, Xu P, Wu D, Li X (2021;Apr 20). Comprehensive evaluation of the efficacy and safety of LPV/r drugs in the treatment of SARS and MERS to provide potential treatment options for COVID-19. Aging (Albany NY).

[13] Ozkavak O O, Tanacan A, Haksever M, Sahin R, Serbetci H, Okutucu G, Aldemir E, Sahin D (2024;Oct 25). The utility of albumin-bilirubin score in patients with intrahepatic cholestasis of pregnancy: A retrospective comparative study. Rev Assoc Med Bras (1992).

[14] Cemortan M, Sagaidac I, Cernetchi O (2025;Apr 8). Comparative analysis of vitamin K levels in women with intrahepatic cholestasis of pregnancy. BMC Pregnancy Childbirth.

[15] Axelsen S M, Schmidt M C, Kampmann U, Grønbæk H, Fuglsang J (2024;Oct). The effect of twin pregnancy in intrahepatic cholestasis of pregnancy: A case control study. Acta Obstet Gynecol Scand.

[16] Wu L, Zhong Y, Wu D, Xu P, Ruan X, Yan J, Liu J, Li X (2022;Sep 10). Immunomodulatory Factor TIM3 of Cytolytic Active Genes Affected the Survival and Prognosis of Lung Adenocarcinoma Patients by Multi-Omics Analysis. Biomedicines.

[17] Pan D, Jiang M, Tao G, Shi J, Song Z, Chen R, Wang D (2024;Dec). The role of Ca2+ signaling and InsP3R in the pathogenesis of intrahepatic cholestasis of pregnancy. J Obstet Gynaecol.

[18] Wu L, Li X, Qian X, Wang S, Liu J, Yan J (2024;Feb 12). Lipid Nanoparticle (LNP) Delivery Carrier-Assisted Targeted Controlled Release mRNA Vaccines in Tumor Immunity. Vaccines (Basel).

[19] Fang Y, Kang Z, Zhang W, Xiang Y, Cheng X, Gui M, Fang D (2024;Aug 10). Core biomarkers analysis benefit for diagnosis on human intrahepatic cholestasis of pregnancy. BMC Pregnancy Childbirth.

[20] Wu L, Liu Q, Ruan X, Luan X, Zhong Y, Liu J, Yan J, Li X (2023;Jun 29). Multiple Omics Analysis of the Role of RBM10 Gene Instability in Immune Regulation and Drug Sensitivity in Patients with Lung Adenocarcinoma (LUAD). Biomedicines.

[21] Ozalp C B, Akdogan S, Cetinavci D, Akin M N, Elbe H, Kasap B (2024;Sep 2). Unveiling the placental secrets: Exploring histopathological changes and TROP2 expression in intrahepatic cholestasis of pregnancy. Placenta.

[22] Şen S H, Şengül M (2023;Jul 7). Could real-time sonoelastography-measured placental strain ratio (PSR) value be a soft marker for the diagnosis of intrahepatic cholestasis of pregnancy? A case-control study and short reviews. Medicine (Baltimore).

[23] Wu L, Zheng Y, Ruan X, Wu D, Xu P, Liu J, Wu D, Li X (2022;Jan 1). Long-chain noncoding ribonucleic acids affect the survival and prognosis of patients with esophageal adenocarcinoma through the autophagy pathway: Construction of a prognostic model. Anticancer Drugs.

[24] Ren Y, Shan X, Ding G, Ai L, Zhu W, Ding Y, Yu F, Chen Y, Wu B (2025;Jan 30). Risk factors and machine learning prediction models for intrahepatic cholestasis of pregnancy. BMC Pregnancy Childbirth.

[25] Tang W Z, Zhao Y F, Wang L, Cai Q Y, Xu W Z, Wen L, Chen X B, Sheng T H, Fan T Q, Liu T H, Li R, Liu S J (2025;Mar 14). Investigating the risks of late preterm and term neonatal morbidity across clinical subtypes of intrahepatic cholestasis of pregnancy. Front Med.

[26] Wu L, Li X, Yan J (2024;Jul). Commentary: Machine learning developed an intratumor heterogeneity signature for predicting prognosis and immunotherapy benefits in cholangiocarcinoma. Transl Oncol.

[27] Ozkan S, Aksan A, Kurt D, Kurt A, Firatligil F B, Sucu S, Reis Y A, Oztürk B G, Çaglar A T (2024;Oct). Are Systemic Inflammation Markers Reliable for Diagnosing Intrahepatic Cholestasis of Pregnancy? A Retrospective Cohort Study. Am J Reprod Immunol.

[28] Wu L, Zhong Y, Yu X, Wu D, Xu P, Lv L, Ruan X, Liu Q, Feng Y, Liu J, Li X (2022;Oct 1). Selective poly adenylation predicts the efficacy of immunotherapy in patients with lung adenocarcinoma by multiple omics research. Anticancer Drugs.

[29] Zhao Y, Zhang Q, Sheng Y, Zhang M, He G, Liu X (2025;May 19). Preterm birth and stillbirth: total bile acid levels in intrahepatic cholestasis of pregnancy and outcomes of twin pregnancies: A retrospective cohort study from 2014 to 2022. BMC Pregnancy Childbirth.

[30] Farisoğullari N, Tanaçan A, Sakcak B, Denizli R, Başaran E, Kara Ö, Yazihan N, Şahin D (2024;Mar). Evaluation of maternal serum vascular endothelial growth factor C and D levels in intrahepatic cholestasis of pregnancy. Int J Gynaecol Obstet.

[31] Wu L, Li H, Liu Y, Fan Z, Xu J, Li N, Qian X, Lin Z, Li X, Yan J (2024). Research progress of 3D-bioprinted functional pancreas and in vitro tumor models. International Journal of Bioprinting.

[32] Cemortan M, Iliadi-Tulbure C, Sagaidac I, Cernetchi O (2024;Mar 10). Assessment of aspartate aminotransferase to Platelet Ratio Index and Fibrosis-4 Index score on women with intrahepatic cholestasis of pregnancy. AJOG Global Reports.

[33] Misra D, Singh N, Faruqi M, Tiwari V, Kumar V, Zafar F (2024;Apr). Evaluating the Utility of Liver Transaminases as Predictors of Feto-Maternal Outcome in Lieu of Serum Bile Acids in Intrahepatic Cholestasis of Pregnancy: A Prospective Observational Study. J Obstet Gynaecol India.

[34] Brenøe J E, van Hoorn E G M, Beck L, Bulthuis M, Bezemer R E, Gordijn S J, Schoots M H, Prins J R (2024;Aug). Altered placental macrophage numbers and subsets in pregnancies complicated with intrahepatic cholestasis of pregnancy (ICP) compared to healthy pregnancies. Placenta.

[35] Wu L, Yang L, Qian X, Hu W, Wang S, Yan J (2024;Aug 16). Mannan-Decorated Lipid Calcium Phosphate Nanoparticle Vaccine Increased the Antitumor Immune Response by Modulating the Tumor Microenvironment. J Funct Biomater.

[36] Köksal Z, Ağbal T, Güçel F, Sarışen Ş (2025;Aug). Relationship Between Serum Autotaxin Levels and Fasting Bile Acid in Intrahepatic Cholestasis of Pregnancy. Am J Reprod Immunol.

